# Improving the Stability of Insulin in Solutions Containing Intestinal Proteases *in Vitro*

**DOI:** 10.3390/ijms9122376

**Published:** 2008-12-01

**Authors:** Liefeng Zhang, Hui Jiang, Wenjie Zhu, Lin Wu, Lingling Song, Qiuyan Wu, Yong Ren

**Affiliations:** Jiangsu Key Laboratory for Supramolecular Medicinal and Applications, College of Life Science, Nanjing Normal University, Nanjing 210097, P.R. China E-Mails: lfzhang1010@yahoo.com.cn (L. Z.); jianghui523@163.com (H. J.); ersaclarke@gmail.com (W. Z.)

**Keywords:** Protection, insulin, degradation, casein, protamine, cyclodextrin

## Abstract

Degradation of insulin was studied in this work. Casein and protamine could obviously suppress degradation of insulin by intestinal enzymes, and could protect insulin from degradation by the mechanism of competition and combination with proteolysis enzyme. What is more, co-incubated with HP-β-CD-casein or HP-β-CD-protamine, most insulin was protected from degradation by intestinal enzymes. In addition, it was found that the complexation of insulin with HP-β-CD was characterized by UV absorption spectra. These results indicated that HP-β-CD, casein and protamine could offer some positive and useful results, and could protect insulin from degradation during their transit through the intestinal tract.

## 1. Introduction

Insulin has been in therapeutic use for almost 80 years, it has been the most effective and durable drug in the treatment of advanced-stage diabetes [1]. Despite significant advancements in the pharmaceutical research field, development of a proper non-invasive insulin delivery system remains a major challenge [2–4]. The oral route represents the most convenient way of drug administration due to high patient compliance and comfort. One of the most important characteristics of oral delivery of peptides and proteins is the need to protect these drugs from proteolytic attack during their transit through the gastrointestinal (GI) tract [5]. Therefore, in order to overcome the enzymatic barriers, various approaches have been developed such as the utilization of enzyme inhibitors [6, 7] and functional polymers [8–10]. However, enzyme inhibitors show several safety problems for clinical applications, a safe and convenient way which can protect insulin from degradation by enzymes in the intestines is an important aspect to be considered.

Casein is the predominant phosphoprotein, accounting for nearly 80% of proteins in milk. Casein consists of a fairly high number of tyrosine (Tyr) residues. Tyr residue is the cleavage site of α-chymotrypsin. At the same time, protamine is an alkaline protein refined from milt. Some 90% of amino acid residues in protamine are arginine (Arg) residues. Arg residue is one of the cleavage sites of trypsin. The cleavage sites of enzymatic recognizability in the two proteins are much more than those in insulin. It was reported that casein could protect other proteins from degradation by trypsin [11–13]. Therefore, casein and protamine were used to evaluate the protection of enzyme degradation in this paper.

Cyclodextrins (CDs) are cyclic oligosaccharides composed of dextrose units joined through α-1,4-glucosidic bonds [14]. They and their derivatives have been widely used in drug delivery application due to their capability of forming complexes with drug molecular [15–17]. These complexes are able to alter the release pattern, increase the solubility and the stability of drugs. CD derivatives, such as HP-β-CD, could prevent insulin from self-aggregating at neutral pH [18]. It also displayed osmotic properties in aqueous solutions depending on their chemical structure and total degree of substitution [19]. Rosa *et al*. [20] reported that the formation of insulin/ HP-β-CD complexes inside microspheres achieved an effective modulation of insulin release rate. In this study HP-β-CD was investigated whether it could increase the stability of insulin.

Taking this information into account, the aim of this study was to combine the virtues of casein and protamine with that of HP-β-CD in order to suppress degradation of insulin by intestinal proteases. In this work, the effect of casein, protamine and HP-β-CD on the protection of insulin from enzyme degradation was studied. Subsequently, co-incubation with HP-β-CD-casein or HP-β-CD-protamine was determined *in vitro*.

## 2. Results and Discussion

### 2.1. Effects of HP-β-CD and casein on degradation of insulin induced by α-chymotrypsin

**Figure 1.**
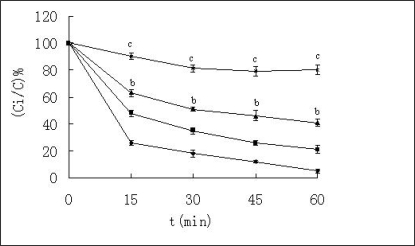
Percentage of the remained insulin against α-chymotrypsin at 37 ºC. ♦-♦ Insulin + α-chymotrypsin; ▪—▪ Insulin + HP-β-CD +α-chymotrypsin; ▴- ▴ Insulin + Casein + α-chymotrypsin; ▪—▪ Insulin + Casein + HP-β-CD +α-chymotrypsin, n=3, mean ± SD. ^b^p < 0.01, ^c^p < 0.001 *vs* insulin + α-chymotrypsin solution according to paired t-test.

**Figure 2.**
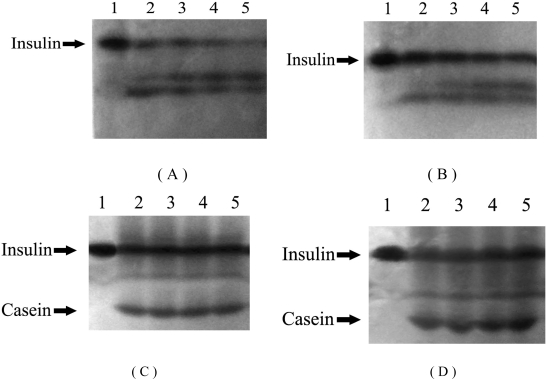
Effects of HP-β-CD and casein on degradation of insulin induced by α-chymotrypsin. Lanes 1-5 in (A) represent insulin incubated with α-chymotrypsin at 37 ºC for 0, 15, 30, 45 and 60 min, respectively; Lanes 1-5 in (B) represent insulin incubated with α-chymotrypsin and HP-β-CD at 37ºC for 0, 15, 30, 45 and 60 min, respectively; Lanes 1-5 in (C) represent insulin incubated with α-chymotrypsin and casein at 37 ºC for 0, 15, 30, 45 and 60 min, respectively; Lanes 1-5 in (D) represent insulin incubated with α-chymotrypsin, HP-β-CD and casein at 37 ºC for 0, 15, 30, 45, 60 min, respectively.

α-Chymotrypsin is a common protease in the intestine, so degradation of insulin by α-chymotrypsin was investigated *in vitro*. The remaining insulin was measured by HPLC (Figure 1) and PAGE (Figure 2). The results using both detection methods were consistent. As shown in Figures 1 and 2, insulin was degraded rapidly by α-chymotrypsin, and most of insulin was degraded after 60 min. In order to examine whether HP-β-CD and casein could protect insulin from degradation or not, HP-β-CD and casein was examined, respectively.

About 25% of the insulin was detected when it was treated with HP-β-CD, and about 45% of the insulin remained undegraded when it was treated with casein. About 80% insulin remained undegraded when it was co-incubated with HP-β-CD and casein. From statistical analysis, it was found that effects of HP-β-CD had no statistical significance (P>0.05), while effects of casein had distinctly statistical significance (P<0.01), and effects of HP-β-CD-casein had very distinctly statistical significance (P<0.001). It indicated that HP-β-CD had little effect, casein had advantage on the protection of insulin, and the two compositions could concurrently protect insulin from degradation by α-chymotrypsin. In addition, the remained insulin against α-chymotrypsin in the solution containing casein and protamine was almost equal to that in the solution containing casein (data not shown). This suggested that protamine had no effect on protecting insulin from degradation by α-chymotrypsin.

### 2.2. Effects of HP-β-CD and protamine on degradation of insulin induced by trypsin

**Figure 3.**
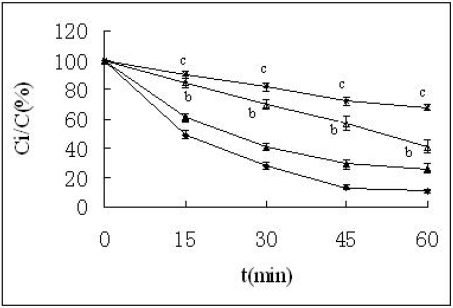
Percentage of the remained insulin against trypsin at 37 ºC. ▪—▪ Insulin + trypsin; ▴-▴ Insulin + HP-β-CD + trypsin; Δ— Δ Insulin + protamine + trypsin; *-* Insulin +protamine + HP-β-CD + trypsin. n=3, mean ± SD. ^b^p < 0.05, ^c^p < 0.01 *vs* insulin + trypsin solution according to paired t-test.

**Figure 4.**
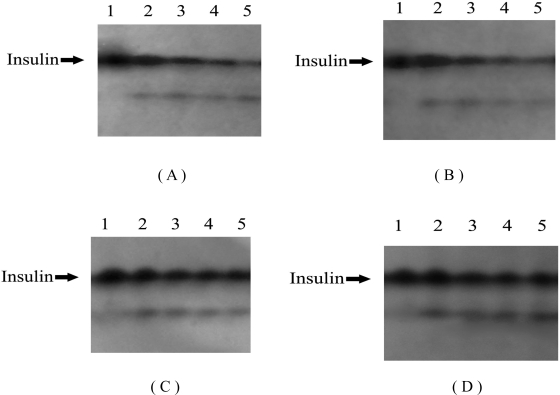
Effects of HP-β-CD and protamine on degradation of insulin induced by trypsin. Lanes 1-5 in (A) represent insulin incubated with trypsin at 37 ºC for 0, 15, 30, 45 and 60 min, respectively; Lanes 1-5 in (B) represent insulin incubated with trypsin and HP-β-CD at 37 ºC for 0, 15, 30, 45 and 60 min, respectively; Lanes 1-5 in (C) represent insulin incubated with trypsin and protamine at 37 ºC for 0, 15, 30, 45 and 60 min, respectively; Lanes 1-5 in (D) represent insulin incubated with trypsin, HP-β-CD and protamine at 37 ºC for 0, 15, 30, 45 and 60 min, respectively.

To further characterize degradation of insulin by the intestinal enzyme, insulin was incubated in the presence of trypsin which was another common protease in the intestine. The results are shown in Figures 3 and 4. The amount of insulin decreased with incubation time from 0 to 60 min, and only about 15% of insulin remained after 60 min. The degradation was partly inhibited when insulin was treated with HP-β-CD, but from statistical analysis it was found that the effectsbof HP-β-CD had no statistical significance (P>0.05). At the same time, insulin degradation was significantly decreased when incubated in the presence of protamine (P<0.05). When insulin was co-incubated with HP-β-CD and protamine, it was shown that about 70% insulin was detected at 60 min (P<0.01), and the amount of remained insulin was significantly increased. In addition, the remaining insulin against trypsin in the solution containing casein and protamine was almost equal to that in the solution containing protamine (data not shown). This suggested that protamine and casein had no concurrent in protecting insulin from degradation by trypsin.

**Figure 5.**
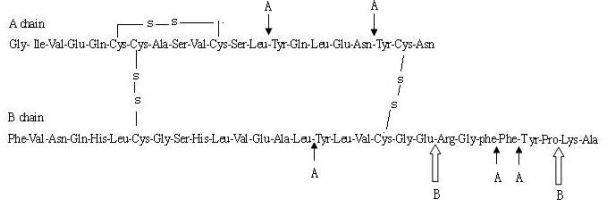
The cleavage site of insulin by proteases. A: The sites are the recognized site of α- chymotrypsin; B : The sites are the recognized sites of trypsin.

Casein and protamine are natural proteins. It was showed that casein and protamine could protect insulin from enzymatic degradation in this study. These results were consistent with the research by Qi *et al*. [21]. As shown in Figure 5, the cleavage sites of insulin by α-chymotrypsin and trypsin were identified [22]. α-Chymotrypsin appeared to cleave initially at the carboxyl side of the B26-Tyr and A19-Tyr residues. In addition, cleavage at B16-Tyr, B25-Phe, and A14-Tyr residues was also happened rapidly. There were four Tyr residues (A14, A19, B16, B26) in the five cleavage sides. The amount of Tyr residues α-chymotrypsin recognized in the casein is much more than those in insulin. At the same time, trypsin cleaved insulin at the B29-Lys and B22-Arg residues. The degradation productions that cleaved in B29-Lys by trypsin had insulin-like activity. However, the desoctapepide cleaved in B22-Arg lost the activity. B22-Arg residue was essential to achieve greater stability without losing activity. More than 90% amino acid residues are Arg residues in protamine. Therefore, casein and protamine could protect insulin from degradation by the mechanism of competition and combination with proteolysis enzymes.

### 2.3. UV absorption spectra of insulin in different solutions

**Figure 6.**
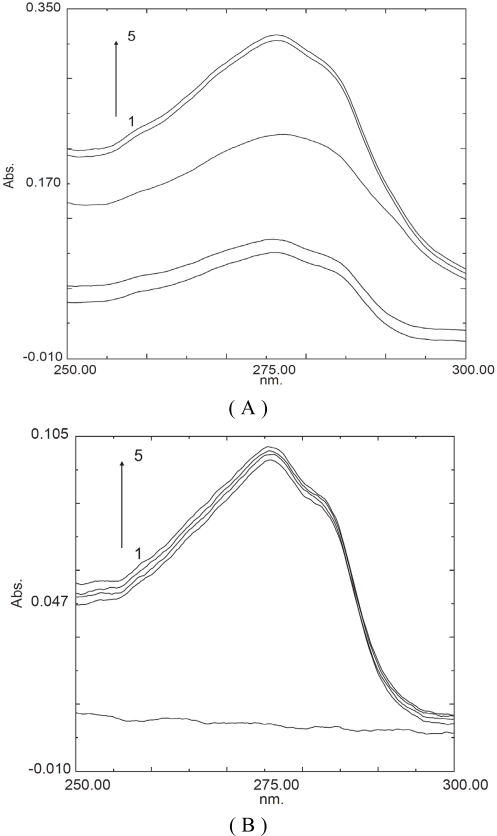
UV absorption spectra of insulin in different solutions. Curves 1-5 in (A) represent insulin, Insulin (100 μg/mL) + HP-β-CD (1 mg/mL), Casein (200 μg/mL), Insulin (100 μg/mL) + Casein (200 μg/mL), Insulin (100 μg/mL)+ Casein (200 μg/mL) + HP-β-CD (1 mg/mL), respectively; Curves 1-5 in (B) represent protamine (200 μg/mL), insulin (100 μg/mL), insulin (100 μg/mL) + protamine (200 μg/mL), insulin (100 μg/mL) + protamine (200 μg/mL) + HP-β-CD (1 mg/mL), insulin (100 μg/mL) + HP-β-CD (1 mg/mL), respectively.

The absorption spectra of insulin in different solutions are shown in Figure 6, Table 1 and Table 2. Adding HP-β-CD to the insulin solution resulted in a significant enhancement of the UV absorption spectra at wavelength from 250 to 300 nm. However, the max. absorption wavelength at 275 nm was not changed. This indicated that the complexation of insulin with HP-β-CD existed. The absorption spectrum of insulin in the solution containing casein was the sum of the absorption spectrum of insulin and that of casein. At the same time, the absorption spectrum of insulin at 275 nm wavelength in the solution containing casein and HP-β-CD was the sum of the absorption spectrum of insulin in the solution containing HP-β-CD and that of casein (Table 1). This indicated that insulin and casein were merely mixed, not complexed. From Figure 6B, it was found that the absorption spectrum of protamine at 275 nm wavelength had not a max. absorption, due to little aromatic amino acids in protamine. The absorption spectrum of insulin in the solution containing protamine was not the sum of the absorption spectrum of insulin and that of protamine. Furthermore, the absorption spectrum of insulin in the solution containing protamine and HP-β-CD was lower than the absorption spectrum of insulin in the solution containing HP-β-CD (Table 2). It suggested that insulin and protamine might be combined. Insulin is with positive charge at pH 7.0 buffer, however, protamine is with negative charge at pH 7.0 buffer. Therefore, insulin and protamine might be integrated.

CDs are well known molecular entities used as pharmaceutical excipients mainly to solubilize and stabilize drugs through complexation [23]. The interaction between insulin and CDs had been studied mostly on absorption enhancement effects through skin, nasal and pulmonary mucus membranes [14, 24]. The UV absorption spectra experiment indicated that the complexation between HP-β-CD and insulin existed. Previously, it was found that β-CD could include insulin easily in neutral water solution in our laboratory [25]. Sajeesh *et al*. [17] and Rosa *et al*. [20] confirmed the formation of HP-β-CD and insulin complexes, using FTIR and fluorescence spectroscopic analysis. Moreover, Dotsidas *et al*. [1] reported that the interaction of insulin with methyl-beta cyclodextrin improves its stability, but Shao *et al*. [26] showed that hydroxypropyl-β-cyclodextrin did neither increase oral bioavailability of insulin, nor it could protect insulin from α-chymotrypsin degradation. In the present study, it was found that HP-β-CD had a little effect of improving the stability of insulin in the intestinal proteases *in vitro,* but the effect had no statistical significance. These results were not entirely consistent. The causes may be correlated with the concentration of proteases.

## 3. Experimental Section

### 3.1. Materials

Insulin (nominal activity: 28 IU/mg) was purchased from Xuzhou Wanbang Biopharma Company (P.R. China), HP-β-CD (MW:1380 Da) was obtained from Sigma Chemical Co. (USA). α-Chymotrypsin (4000 U/mg), trypsin (25,000 U/g), casein and protamine were purchased from Shanghai Biochemistry Pharma Company (P.R. China). Acetonitrile was HPLC grade, and all other reagents used were of analytical grade and commercially available.

### 3.2. HPLC method

HPLC was carried out according to Todo *et al.* [27] with minor modifications. Analyses of remaining insulin were carried out by reserve phase HPLC with an isocratic system (Shimadzu Co., Kyoto) using the raw insulin bulk drug as the standard (28 IU/mg). The HPLC system was composed of a pump (LC-10ADvp), diode array detector (SPD-M10Avp), column oven (CTO-10ASvp), and LC workstation (CLASS-LC10). The mobile phase was a 72:28 mixture of 0.2 mol/L sodium sulphate buffer (pH 3.0) and acetonitrile at a flow rate of 1.0 mL/min. The column was a Shodex Asahipak ODP-50 6D (4.6mm × 150 mm, 5 um) (Showa Denko, Ltd., Tokyo) heated at room temperature. Ultraviolet absorption was measured at 214 nm.

### 3.3. Polyacrylamide gel electrophoresis (PAGE)

PAGE was carried out according to Laemmli [28] with minor modifications. The running gel, stacking gel and electrode buffer are without sodium dodecyl sulfate (SDS). The running gel and stacking gel contained 15% and 4.5% acrylamide, respectively. After electrophoresis, the gels were subjected to stain with Coomassie Brilliant R-250.

### 3.4. Degradation of insulin induced by α-chymotrypsin and trypsin

Insulin was dissolved in a few drops of 0.05 mol/L HCL, then 0.05 mol/L PBS (phosphate buffer, pH=7.0) was added. α-Chymotrypsin (5 mg/mL) and trypsin (12 mg/mL) solutions were prepared with Tris-HCL buffer (pH=8.0), respectively. Casein (5 g) was dissolved in 0.1 mol/L NaOH (10 mL), then was diluted to 100 mL with PBS (pH=7.0), The pH of final concentration was again measured and adjusted to pH 7.0. 5% Protamine solution was prepared with PBS (pH=7.0). Insulin solution and enzyme solutions were incubated at 37 ºC for 15 min. Different treatments were administered. The final concentration of insulin in the solution was 0.5 mg/mL. The final mass ratios in the solution were as following, insulin:HP-β-CD = 1 :30; insulin:casein = 1:10; insulin:protamine = 1:10; insulin:casein or protamine:HP-β-CD = 1:10:30. Before the addition of a-chymotrypsin solution, insulin solution was vortexed at high speed for 5 s and a sample was withdrawn and added with ice-cold 0.5% TFA acid as the zero time sample. Samples were withdrawn for 15, 30, 45 and 60 min after the addition of the enzyme solution at 37 ºC. The final enzyme concentrations of α-chymotrypsin and trypsin were 8 mg/L [29] and 600 mg/L [21], respectively. Then the samples were analyzed by PAGE and HPLC after adding 50 μl of ice-cold 0.5% TFA acid to terminate the reaction. Every experiment was carried out three times. The average and the standard deviation of the data were calculated.

### 3.5. Spectroscopic methods

Absorption spectra were recorded on a Shimadzu UV-2450 spectrophotometer. The absorption spectra were carried out according to Kano *et al*. [30].

## 4. Conclusions

Insulin is ineffective by oral administration due to the low stability in the GI and their poor absorption [31]. Therefore, it is essential for oral insulin delivery system to protect insulin from enzymatic degradation. In this study, it was also found that insulin was very sensitive to these proteases in the intestine. Furthermore, casein and protamine could partly suppress degradation of insulin in the intestinal proteases *in vitro*, and could protect insulin from degradation by the mechanism of competition and combination with proteolysis enzymes. In addition, it was found that the complexation of insulin with HP-β-CD was characterized by UV absorption spectra. When the complexes were co-incubated with casein or protamine, most insulin was protected from the enzymatic degradation. In addition, HP-β-CD and casein had synergic effects on protection of insulin from enzymatic degradation.

Casein and protamine which are esculent and nontoxic proteins had obvious advantages in protecting insulin from enzymatic degradation. At the same time, HP-β-CD is also a safe drug additive. The present study combined the virtues of casein and protamine with that of HP-β-CD to protect insulin from enzymatic degradation. Taken together, HP-β-CD, casein and protamine could offer some positive and useful results, and could be an efficient way to achieve the oral delivery of insulin.

## Figures and Tables

**Table 1.** The absorption of insulin and casein.

Component in the solution	ΔA_275_
Insulin	0.098
Insulin + HP-β-CD	0.102
Casein	0.219
Insulin + Casein	0.317
Insulin + Casein + HP-β-CD	0.321

**Table 2.** The absorption of insulin and protamine.

Component in the solution	ΔA_275_
protamine	0.006
Insulin	0.096
Insulin + protamine	0.098
Insulin+protamine+HP-β-CD	0.099
Insulin + HP-β-CD	0.101
